# A structured laughter yoga therapy program on patients with chemotherapy-induced nausea and vomiting: A randomized clinical trial

**DOI:** 10.1016/j.apjon.2023.100337

**Published:** 2023-11-15

**Authors:** Mohammad Namazinia, Seyyed Reza Mazlum, Samira Mohajer, Khatijah Lim Abdullah, Maryam Salehian

**Affiliations:** a Department of Nursing, School of Nursing and Midwifery, Torbat Heydariyeh University of Medical Sciences, Torbat Heydariyeh, Iran; b Department of Medical Surgical Nursing, School of Nursing and Midwifery, Mashhad University of Medical Sciences, Mashhad, Iran; c Nursing and Midwifery Care Research Center, Mashhad University of Medical Sciences, Mashhad, Iran; d Department of Nursing, School of Medical and Life Science, Sunway University, Bandar Sunway, Malaysia

**Keywords:** Laughter yoga, Quality of life, Cancer patients laughter yoga, Chemotherapy, Cancer, Nausea and vomiting

## Abstract

**Objetive:**

Chemotherapy is a prevalent cancer treatment, often accompanied by debilitating side effects such as nausea and vomiting. This study explores the potential effectiveness of laughter yoga, a combination of exercise and voluntary laughter, in alleviating chemotherapy-induced nausea and vomiting.

**Methods:**

This two-group randomized clinical trial was conducted on 69 cancer patients undergoing chemotherapy at the Reza Chemotherapy and Oncology Center, Mashhad, Iran, in 2018. Patients were randomly divided into intervention and control groups. Both groups received routine self-care training, with the addition of four 20-min to 30-min laughter yoga sessions held immediately before one of their chemotherapy appointments for the intervention group only. Nausea and vomiting were assessed using the Morrow Assessment of Nausea and Emesis questionnaire at two stages, before and after the intervention. Data were analyzed with Chi-square, Independent-t, Mann–Whitney, Wilcoxon, and McNemar tests using Statistical Package for the Social Sciences (SPSS).

**Results:**

The mean age of patients in the intervention group was 49.0 ± 9.6 years, while in the control group, it was 45.2 ± 12.6 years. The intragroup comparison showed a statistically significant decrease in the severity and duration of nausea in the intervention group and a statistically significant increase in the severity and duration of nausea in the control group from pre-test to post-test (*P* < 0.05). The intergroup comparison showed no statistically significant difference between the two groups in terms of vomiting conditions.

**Conclusions:**

Laughter yoga demonstrates promise in improving chemotherapy-induced nausea, suggesting its potential recommendation for managing this distressing side effect. Further research is warranted to explore its broader application in cancer care.

**Trial registration:**

This study (No. IRCT20180429039463N1) was registered in the Iranian Registry of Clinical Trials on 21/08/2018.

## Introduction

Cancer is the cause of 9% of deaths worldwide.^1^^,^^2^ It is the second leading cause of death in developing countries after cardiovascular diseases and the third leading cause of death in Iran.^3^ In 2018, about 18 million new cancer cases and nearly 10 million cancer deaths were reported worldwide.^4^ Due to the increase in population growth and the aging population, the number of cancer cases is likely to rise faster after 2030.^5^^,^^6^

For many cancer patients, one definitive treatment for improving life expectancy and survival is chemotherapy^7^. Chemotherapy involves administering drugs to kill tumor cells by disrupting their functions and reproduction^8^ and is commonly used in the treatment of a wide variety of cancers.^7^ The most common side effects of chemotherapy drugs are nausea and vomiting.^9^ One of the most important causes of chemotherapy-induced nausea and vomiting is the activation of the chemoreceptor trigger zone by chemotherapy substances.^10^ It has been estimated that 48%–70% of chemotherapy patients suffer from nausea and vomiting despite receiving antinausea and vomiting drugs.^9^

Chemotherapy-induced nausea and vomiting can be managed by several pharmacological and nonpharmacological treatments.^11^ This condition can be controlled by a variety of drugs, including serotonin receptor antagonists, dexamethasone, neurokinin antagonists, and metoclopramide.^12^ Although pharmacological treatments reduce nausea and vomiting, they do not completely eliminate it, which is why about 61% of patients still complain about this condition.^9^ Furthermore, these drugs are expensive and can have serious side effects such as extrapyramidal effects, hypotension, headache, constipation, fatigue, dry mouth, dizziness, diarrhea, and restlessness, which greatly limit their use.^13^^,^^14^ Therefore, many studies recommend using nonpharmacological techniques such as listening to music, relaxation techniques, acupressure, acupuncture, and yoga to reduce chemotherapy-induced nausea and vomiting.^11^ These techniques can be performed by patients with simple tools without any assistance, which helps patients remain independent. Furthermore, they are easily accepted by all patients and do not have the negative side effects and consequences of drug interventions.^11^^,^^14^^,^^15^ Research has shown that most patients have a positive attitude towards care practices that are available outside the hospital, i.e., those that fall in the category of complementary medicine.^16^, ^17^, ^18^, ^19^, ^20^ There is a growing belief in the effectiveness of complementary therapies as secondary treatment and no study has reported any serious side effects for these therapies.^21^

Laughter yoga is a form of supportive complementary therapy that involves performing a variety of exercises in combination with laughter. This treatment combines yoga breathing and stretching exercises with unconditional laughter, i.e., laughter that is not triggered by jokes or comedy.^22^^,^^23^ Structural Yoga Therapy is a therapeutic modality that seeks to alleviate structural problems and diseases by adapting yoga poses to the individual's unique needs. It respects the body's innate capacity to recognize safe, healthy movement and healing. When health is optimal, there is a natural balance of muscle strength and range of motion. But illness, injury, and structural anomalies can disrupt that harmony.

Laughter yoga is done in groups because the experience of laughing in a group environment provides more positive emotions and helps people improve their communication skills.^24^^,^^25^ This type of yoga can reduce anxiety and stress and improve mental health.^26^ It is also an easy, inexpensive, and highly accessible method for maintaining and promoting the health of patients.^27^ The results of a study by Armat et al. 2022 have shown that laughter yoga can reduce depression and anxiety in elderly women and help increase their quality of life.^28^ Farifteh et al. have also reported that laughter yoga can reduce the stress of cancer patients before undergoing chemotherapy.^29^ Also, several studies have shown the positive effects of laughter yoga.^30^, ^31^, ^32^ Since many of the mechanisms that exacerbate chemotherapy-induced nausea and vomiting are related to stress or anxiety, and reducing anxiety and stress can increase a cancer patient's satisfaction with life, a stress/anxiety reduction practice like laughter yoga could be effective in reducing chemotherapy-induced nausea and vomiting. Despite the potentially beneficial effects of structured laughter yoga as a supportive care program, limited studies have been conducted in this field. Therefore, this study aimed to examine the effectiveness of structured laughter yoga as a supportive care program for nausea and vomiting in cancer patients undergoing chemotherapy. Thus, this study attempted to determine whether laughter yoga can reduce nausea and vomiting in patients undergoing chemotherapy.

## Methods

### Setting and study design

This study was designed as a randomized controlled clinical trial and performed from October 2018 to June 2019 on patients undergoing chemotherapy at the Imam Reza Chemotherapy and Oncology Center in Mashhad, Iran, which is the largest and best-equipped chemotherapy and radiotherapy center in the east of Iran.

### Inclusion and exclusion criteria

The inclusion criteria were: age 18–60 years, fully conscious, history of nausea and vomiting, having non-metastatic cancer (based on diagnostic tests, symptoms, and clinical examinations and the approval of an oncology doctor), no auditory or visual problems, undergoing four sessions of chemotherapy per month, no symptoms of osteomyelitis, no upper gastrointestinal cancer, not undergoing radiotherapy simultaneously, and have the mental and physical capability to perform laughter yoga exercises. The exclusion criteria were those experiencing major stress (with the approval of the psychologist of the chemotherapy center), exacerbation of the disease, the need for intensive care, any change in the chemotherapy program due to thrombocytopenia, and any change in the chemotherapy regimen.

### Measurement instruments

Data collection tools were demographic information questionnaires and the Morrow Assessment of Nausea and Emesis (MANE) questionnaire. The MANE questionnaire assesses the frequency, duration, and severity of nausea and vomiting before, during, and after a treatment on a 7-point Likert scale with scores from 0 to 6, with 0 indicating the absence of nausea and vomiting and 6 indicating intolerable nausea and vomiting. The 16 items of this questionnaire can be used to rate the occurrence of nausea and vomiting, describe its nature, duration, and time of occurrence relative to the time of chemotherapy, and describe its worst-case condition.^33^

This tool included questions about the incidence of nausea during or after chemotherapy, duration of nausea, severity of nausea, vomiting during or after chemotherapy, duration of vomiting, severity of vomiting, use of antinausea and vomiting medication, and the effect of medication.^34^

The reliability of the MANE questionnaire has been confirmed in studies conducted in Iran and other countries with a Cronbach's alpha coefficient of 0.61–0.78.^35^, ^36^, ^37^ After getting permission, the MANE questionnaire was translated into Persian by the research team. The translation was given to experts in the English language. Two main translated versions were compared after re-translation to English. The translation was confirmed. The validity of the demographic information questionnaire and the MANE questionnaire was established using the qualitative content validity method. For this purpose, the tool was provided to 10 people including university professors and others with expertise on the subject, for evaluation and finalized by applying their recommended corrections and modifications. For reliability assessment, the questionnaires were administered to 10 participants, and internal consistency was measured by Cronbach's alpha, which was determined to be 0.81.

MANE questionnaire were completed before and after the laughter yoga sessions by the cancer patients (CPs) through interviews in a quiet room at the meeting hall next to the Chemotherapy Center.

### Sample size and randomization

Since no similar study was found that examined the effect of laughter yoga on the variables of nausea and vomiting, the sample size was based on the results of a pilot study on 10 patients from the research unit in each group using two formulas to compare the averages and comparisons of proportions was estimated, and the highest number obtained was considered the sample size of this research. The final sample size was estimated with a confidence level of 95% and a test power of 80% equal to 34 patients in each group, and was related to the incidence of nausea.

$$N={\left(Z_{1-\alpha /2}+Z_{1-\beta }\right)}^{2}\;{\left(p_{1}\;\left(1-p_{1}\right)\;p_{2}\;\left(1-p_{2}\;\right)/\;\left(p_{1}-p_{2}\right)\right)}^{2}$$

$$Z_{1-\alpha /2}=196$$

$$Z_{1-\beta }=0.85$$

$$P_{1}=0.273$$

$$P_{2}=0.591$$

**Fig. 1:**
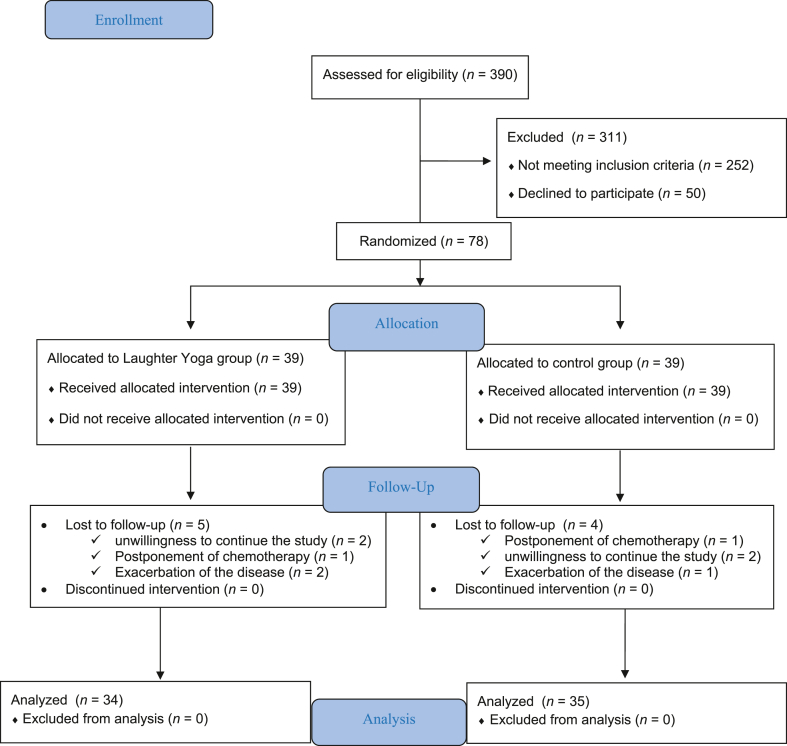
The CONSORT checklist of study.

To compensate for any dropout rate among the two groups, the study was conducted on 38 people per group to account for 10% of the sample loss. Of these 76 people, 4 in the intervention group and 3 in the control group were excluded from the study, leaving 34 eligible subjects in the intervention group and 35 in the control group (Fig. 1).

Patients were chosen continuously and purposefully and were divided into two groups randomly. For this purpose, eligible patients were assigned to the intervention and control groups based on a random sequence generated by the Statistical Package for the Social Sciences (SPSS) software that was kept in a closed envelope. After receiving an explanation about the purpose and method of the research, patients who wished to participate in the study were asked to sign a written informed consent form.

### The intervention protocol of structured laughter yoga

The first author of the study referred to the chemotherapy department in the Imam Reza Chemotherapy and Oncology Center in Mashhad and identified patients with conditions for entering the study. In this study, 390 patients were evaluated for eligibility.

In this study, the structured laughter yoga program was a supportive care approach. At the beginning of the intervention, in the orientation session, the first author of the article, who was the supportive care coordinator, explained the intervention program to the intervention group and guided the cancer patients during the program. This intervention was provided by this researcher, who has completed the laughter yoga training course under the supervision of the professional laughter yoga instructor. During the 16-hour period, the researcher found the necessary skills to implement the program. The study questionnaires were completed before and after the laughter yoga sessions by the CPs through interviews in a quiet room at the meeting hall next to the Chemotherapy Center. The intervention group received the structure laughter yoga exercises as supportive care for four sessions, each lasting 20–30 min, and each blast of laughter took about 30–45 min. Each session was conducted at one-week intervals.

Laughter yoga exercises were held in three groups of 8, 12, and 14 CPs. The intervention was carried out before the chemotherapy according to the protocol. In our study, Structural Yoga Therapy was designed by the professional laughter yoga instructor and performed by a trained supportive care coordinator in a standing position following the 15 steps of the structure laughter yoga protocol, which consists of four sessions of the intervention lasting between 20 and 30 minutes (Supplementary material 1).

### Control

For people in the control group, the researcher only provided routine self-care training at the center's meeting hall, which was located next to the chemotherapy ward. This training was in the form of one session of face-to-face training with educational pamphlets. The exact same training was also provided for the intervention group. Educational content was compiled and prepared by reviewing the latest resources including reference books, articles, and the national cancer prevention and control plan developed by the Iranian Ministry of Health and the World Health Organization, under the supervision of the research team's advising and supervising professors, who specialize in the education and care of cancer patients undergoing chemotherapy and the health psychology of cancer patients.

### Data analysis

Statistical analyses were performed using Statistical Package for the Social Sciences (SPSS) version 20. Descriptive statistics (frequency distribution, mean, and standard deviation) were used to describe and categorize the data, and inferential statistics including Chi-square and Mann–Whitney tests, were used to test the hypothesis. Inter-group comparisons were made using the Wilcoxon and McNemar tests. The normality of quantitative variables was assessed by the Kolmogorov–Smirnov test, which showed the non-normal distribution of data. For all tests, the significance level was considered to be 0.05.

## Results

Females constituted the majority of patients in both groups, specifically 64.7% (*n* = 22) in the intervention group and 68.6% (*n* = 24) in the control group. In the intervention group, 47.1% (*n* = 16) and in the control group, 31.4% (*n* = 11) had gastrointestinal cancers. The majority of patients, 91.2% (*n* = 31) in the intervention group and 97.1% (*n* = 34) in the control group, had prior experience of chemotherapy. The majority of patients in the intervention group (97.1%, *n* = 33) and in the control group (100.0%, *n* = 35) had not experienced laughter yoga. Statistical tests (*P* > 0.05) showed the homogeneity of the two groups in terms of age, gender, type of cancer, previous chemotherapy experience, and previous experience of laughter yoga (Table 1).

**Table 1:** Demographic variables of the intervention and control groups.

Variable	Group		*P* value
	Intervention (*n* = 34)	Control (*n* = 35)	
Age (years, mean ± SD)	49.0 ± 9.6	45.2 ± 12.6	0.378
Frequency of chemotherapy (mean ± SD)	6.3 ± 6.8	5.5 ± 4.6	0.871
Gender, *n* (%)			
Male	12 (35.3)	11 (31.4)	0.733
Female	22 (64.7)	24 (68.6)	
Income, *n* (%)			
Less than enough	22 (64.7)	21 (60.0)	0.598
Eenough	9 (26.5)	13 (37.1)	
More than enough	2 (5.9)	1 (2.9)	
Tumor, *n* (%)			
Gastrointestinal	16 (47.1)	11 (31.4)	0.505
Breast	11 (32.4)	10 (28.6)	
Lung	3 (8.8)	5 (14.3)	
Genital	2 (5.9)	5 (14.3)	
Lymphatic	0 (0.0)	2 (5.7)	
Bone	2 (5.9)	2 (5.7)	
Marital status, *n* (%)			
Single	2 (5.9)	4 (11.4)	0.673
Married	31 (91.2)	31 (88.6)	
Other	1 (2.9)	0 (0.0)	
Previous chemotherapy experience, *n* (%)			
Yes	31 (91.2)	34 (97.1)	0.298
No	3 (8.8)	1 (2.9)	
Experience laughing yoga, *n* (%)			
Yes	1 (2.9)	0 (0.0)	0.493
No	33 (97.1)	35 (100.0)	
Family history of cancer, *n* (%)			
Yes	14 (41.2)	7 (20.0)	0.056
No	20 (58.8)	28 (80.0)	

Frequency of chemotherapy: Number of previous chemotherapy sessions; Previous chemotherapy experience: historical treatment of chemotherapy.

After analyzing the results of the MANE questionnaire, the results showed no statistically significant difference between the two groups in terms of the frequency of nausea, neither at the pre-test nor at the post-test (*P* > 0.05). In the pre-test, the intervention group, 35.3% (*n* = 12) and in the control group, 54.3% (*n* = 19) had no nausea. In the post-test, the intervention group 50.0% (*n* = 17) and the control group 42.9% (*n* = 15) had no nausea. The intragroup comparison also showed no statistically significant change in this parameter in any of the groups (*P* > 0.05). However, the intragroup comparison showed a statistically significant decrease in the severity of nausea in the intervention group from pre-test to post-test (*P* = 0.020) and a statistically significant increase in the severity of nausea in the control group from pre-test to post-test (*P* = 0.038). In the inter-group comparison, the intervention and control groups were not statistically different in terms of the mean duration of nausea in any of the stages (*P* > 0.05). However, the intragroup comparison showed a statistically significant decrease in the duration of nausea in the intervention group from pre-test to post-test (*P* = 0.019) and a statistically significant increase in the duration of nausea in the control group from pre-test to post-test (*P* = 0.022) (Table 2).

**Table 2:** Comparison of nausea before and after the intervention in the intervention and control groups.

Nausea	Group				*P* value
	Intervention (*n* = 34)		Control (*n* = 35)		
	Pretest	Posttest	Pretest	Posttest	
Frequency, *n* (%)					
Yes	22 (64.7)	17 (50.0)	16 (45.7)	20 (57.1)	Before the intervention *P* = 0.113 After the intervention *P* = 0.522
No	12 (35.3)	17 (50.0)	19 (54.3)	15 (42.9)	
Intragroup comparison	*P* = 0.227		*P* = 0.219		
Severity, *n* (%)					
No vomiting	12 (35.3)	21 (61.8)	19 (54.3)	18 (51.4)	Before the intervention *P* = 0.161 After the intervention *P* = 0.121
Very mild	10 (29.4)	3 (8.8)	8 (22.9)	1 (2.9)	
Mild	4 (11.8)	7 (20.6)	2 (5.7)	4 (11.4)	
Moderate	5 (14.7)	2 (5.9)	2 (5.7)	8 (22.9)	
Severe	3 (8.8)	1 (2.9)	4 (11.4)	4 (11.4)	
Intragroup comparison	*P* = 0.020		*P* = 0.038		
Duration (hours), Mean ± SD	11.2 ± 5.0	8.1 ± 4.7	14.3 ± 3.4	20.3 ± 8.3	Before the intervention *P* = 0.264 After the intervention *P* = 0.284
Intragroup comparison	*P* = 0.019		*P* = 0.022		

There was no statistically significant difference between the intervention and control groups in the pre-test and post-test stages in terms of the frequency of vomiting (*P* > 0.05). The intragroup comparison also showed no statistically significant change in this parameter in any of the groups (*P* > 0.05). The results also showed no statistically significant difference between the two groups in terms of the severity of vomiting, neither at the pre-test nor at the post-test stage (*P* > 0.05), and no statistically significant change in this parameter from pre-test to post-test in any of the groups (*P* > 0.05). There was no statistically significant difference between the two groups in terms of the duration of vomiting, neither at the pre-test nor at the post-test stage (*P* > 0.05), and the intragroup comparison showed no statistically significant change in this parameter from pre-test to post-test in any of the groups (*P* > 0.05) (Table 3).

**Table 3:** Comparison of vomiting before and after the intervention in the intervention and control groups.

Vomiting	Group				*P* value
	Intervention (*n* = 34)		Control (*n* = 35)		
	Pretest	Posttest	Pretest	Posttest	
Frequency, *n* (%)					
Yes	2 (5.9)	1 (2.9)	2 (5.7)	1 (2.9)	Before the intervention *P* = 0.976 After the intervention *P* = 0.983
No	32 (94.1)	33 (97.1)	33 (94.3)	34 (97.1)	
Intragroup comparison	*P* = 1.000		*P* = 1.000		
Severity, *n* (%)					
No vomiting	32 (94.1)	33 (97.1)	33 (94.3)	35 (100.0)	Before the intervention *P* = 0.953 After the intervention *P* = 0.310
Very mild	0 (0.0)	0 (0.0)	1 (2.9)	0 (0.0)	
Mild	1 (2.9)	0 (0.0)	0 (0.0)	0 (0.0)	
Moderate	0 (0.0)	1 (2.9)	1 (2.9)	0 (0.0)	
Severe	1 (2.9)	0 (0.0)	0 (0.0)	0 (0.0)	
Intragroup comparison	*P* = 0.180		*P* = 0.180		
Duration (hours), Mean ± SD	0.0 ± 9.3	0.0 ± 4.2	0.1 ± 29.2	0.0 ± 1.4	Before the intervention *P* = 0.988 After the intervention *P* = 0.640
Intragroup comparison	*P* = 0.157		*P* = 0.715		

## Discussion

This study investigated the effect of a structured laughter yoga program as a supportive care approach on nausea and vomiting in cancer patients undergoing chemotherapy. The findings showed that the implemented laughter yoga program improved the severity and duration of nausea in patients undergoing chemotherapy but had no effect on the vomiting of these patients. Since the review of the literature did not reveal any similar study on the effect of laughter yoga on nausea and vomiting in patients undergoing chemotherapy, the results of studies that reported effectiveness other complementary therapies and nonpharmacological methods were used for comparison.

### The effect of the structured laughter yoga therapy program on nausea in patients undergoing chemotherapy

In a study by Ragwandra et al., a yoga program was able to reduce the severity of nausea in patients undergoing chemotherapy, which is consistent with the findings of the present study.^35^ It can be debated that laughter is a muscle relaxation technique; thus, research investigating the relationship between muscle relaxation techniques and nausea and vomiting seems to support these study findings.^25^ In a study by Wang et al., electrical stimulation could improve the severity of nausea in patients undergoing chemotherapy,^38^ which is consistent with our findings. Electrical stimulation is similar to the intervention in the present study in terms of reliability, noninvasiveness, safety, and nontoxicity. Moreover, in some studies, electrical stimulation has been able to reduce anxiety and trigger changes in physiological parameters.^39^^,^^40^ Therefore, this is consistent with the results of the present study, as both of these methods are complementary and nonpharmacological. In a study by Tikisar et al., music therapy and guided visual imagery were able to improve the duration of nausea in patients undergoing chemotherapy.^41^ According to some studies, there is a significant relationship between stress and nausea in the sense that stress prolongs the duration of nausea.^6^^,^^42^ This can explain the similarity between the aforementioned results and our results in terms of the effect on the duration of nausea, despite the difference in the type of intervention. However, a study by Reed et al. reported that yoga had no effect on the duration of nausea in patients with breast cancer.^43^ It is proposed that the addition of laughter to yoga in this study have help reduce the duration of nausea in patients undergoing chemotherapy. Moreover, the short duration of yoga sessions held in Reed's study could have affected the duration of nausea.^31^^,^^44^ Also, in a study by Taniha et al., yoga was able to improve the incidence of nausea in patients with irritable bowel syndrome.^45^ In the present study, however, laughter yoga had no such effect on nausea. This discrepancy could be due to the difference between patients with irritable bowel syndrome and those undergoing chemotherapy in terms of the mechanism of nausea.

### The effect of the structured laughter yoga therapy program on vomiting in patients undergoing chemotherapy

The findings of this study showed that the implemented laughter yoga program had no effect on the vomiting of these patients. In a study by de Carvalho et al., muscle relaxation could not reduce the incidence of vomiting in patients undergoing chemotherapy.^46^ Other studies have also reported that short-term relaxation has no effect on vomiting.^47^ In a study by Volsterling et al., only a few people in the control and relaxation groups reported vomiting and nausea, which means the findings must be validated by further research on a greater number of people.^48^ The ineffectiveness of laughter yoga on vomiting in the present study could also be due to the lower prevalence of this condition compared to nausea. A study by Taspinar et al. also found that acupressure had no effect on the severity of vomiting in patients undergoing chemotherapy.^49^ The reason for this agreement could be the limited number of people with vomiting in both studies, which makes it difficult to find a statistically significant difference between the groups. In a study by Moradian et al., a music program could not reduce the severity of vomiting in women with breast cancer,^50^ which is consistent with the results of the present study. Ordinary music is known to reduce stress and anxiety, which makes it similar to yoga in this respect. In this case, the low incidence of vomiting in both studies could be the reason for not finding a significant difference between the two groups. But in the study of Tikisar et al., music therapy and guided visual imagery were able to reduce the duration of vomiting.^41^ Despite some similarities between the interventions of the present study and those of Tikisar et al., there is an inconsistency between these findings. One of the reasons for this discrepancy is that the duration of chemotherapy, which was 30–90 min in Tikisar's study but in the present study based on the chemotherapy protocols, was 3–4 h. As a result, the side effects of chemotherapy including nausea and vomiting in the present study were more severe than in Tikisar's study. Furthermore, in Tikisar's study, music therapy and guided visual imagery were performed only once before chemotherapy, and the effect was measured immediately after the intervention. Therefore, the reported results are probably related to the immediate effects of the intervention and may not reflect an effect on the duration of vomiting in the long run.

By activating the neural pathways of emotions such as joy and mirth, laughter can improve the mood and make patients' physical and emotional responses to stress less intense. The findings of our study showed that the addition of laughter to yoga in this study helped reduce nausea and vomiting in cancer patients undergoing chemotherapy. Moreover, the results of our study suggest that laughter yoga could be feasible at supportive care facilities, and patients and caregivers would like to continue with similar practices. Our data provide preliminary evidence that laughter yoga could be effective in reducing some of the side effects of cancer treatment, such as nausea and vomiting, which could be used to improve the quality of life of cancer patients. Thus, the findings of our study presented the distinctive features of laughter yoga that distinguish it as a valuable addition to the field.^31^^,^^51^

### Limitations

This study had several limitations. First, the program was limited to only four sessions, which means prolonging the duration of the program may change the results. Second, laughter yoga may cause a social-desirability bias, which may affect participants’ responses. Third, this study was conducted with a small sample size and at one oncology center in Iran, which may limit generalizability to other parts of the country. Therefore, further studies are proposed to verify the generalizability of the findings by including more research sites and larger samples. Third, since some patients and their families were concerned about participating in the laughter yoga sessions, some family members were insistent on being present during the laughter yoga sessions, which could have affected the findings. The researchers tried to alleviate these concerns by explaining the process as much as needed and acquiring a certificate from the treating physician for performing laughter yoga. Lastly, the stark disparity between the findings of this study and those from Western countries underscored the need to be cautious about the positive results. Thus, the current study needs to be replicated and validated using objective measures of these constructs.

### Implications for nursing practice and research

Considering the importance of the educational and caring role of nurses and its significant impact on improving the quality of services provided to cancer patients, and on the other hand, to improve the nausea of the studied patients in the intervention group, laughter yoga can be used as an accessible, effective, and low-cost approach in different hospital departments. Also, many of the mechanisms that exacerbate chemotherapy-induced nausea and vomiting are related to stress or anxiety, and reducing anxiety and stress can increase a cancer patient's satisfaction with life. A stress and anxiety reduction practice like laughter yoga could be effective in reducing chemotherapy-induced nausea and vomiting.

## Conclusions

Although there are some disparities between this study's findings and those from other countries, the structured laughter yoga program was a supportive care approach, provided new insights in understanding the potential effects of nonpharmacological interventions on treatment-related concerns such as nausea and vomiting. This study shows the possibilities of using laughter yoga to improve the nausea of cancer patients undergoing chemotherapy. Laughter yoga is probably associated with reduced stress and anxiety and an improved positive mood, which in turn improves nausea. However, laughter yoga showed no effect on vomiting, a finding that could be due to the low frequency of vomiting in this study's patients. Therefore, more research should be conducted on the exact mechanism of the effect of a structured laughter yoga program as supportive care on nausea and vomiting. Future studies are recommended to try to obtain more accurate results regarding the effect of laughter yoga by prolonging the duration of the intervention.

## Acknowledgments

The researchers would like to thank nurses, patients, and the families who collaborated in this study.

## CRediT author statement

Mohammad Namazinia, Methodology design, prepared the writing of the initial draft, acquisition of data, analyze and interpret the data, conceptualize the paper, and review and synthesize the literature. Seyed Reza Mazloum, Methodology design, obtained funding for the manuscript, supervised, proof-read, and provided intellectual support in terms of statistical analysis and administrative, technical, and material support and supervised in the preparation of the manuscript. Samira Mohajer, Methodology design, supervised, proof-read, and provided intellectual support in terms of administrative, technical, and material support and supervised in the preparation of the manuscript. Khatijah Lim Abdullah, Methodology design, provided critical review and significant revision of the manuscript for important intellectual content, proof-read, and supervised the preparation of the manuscript. Maryam Salehian, provided critical review. All authors were granted complete access to all the data in the study, with the corresponding authors bearing the final responsibility for the decision to submit for publication. The corresponding authors affirm that all listed authors fulfill the authorship criteria and that no others meeting the criteria have been omitted.

## Declaration of competing interest

All authors have none to declare.

## Funding

This study was under the financial aegis of Research Deputy of Mashhad University of Medical Sciences, Mashhad, Iran (Grant No. 970132). The funders had no role in considering the study design or in the collection, analysis, interpretation of data, writing of the report, or decision to submit the article for publication.

## Ethics statement

The study was conducted after receiving approval from the regional research ethics committee with the code IR.MUMS.NURSE.REC.1397.021, and registered in the Iranian Registry of Clinical Trials with the code IRCT20180429039463N1. The research plan was presented to relevant officials in the chemotherapy ward of Imam Reza Chemotherapy and Oncology Center, and coordinated with the center's managers and the head of the ward. All patients in both groups entered the study after receiving an oral face-to-face explanation about the research by the researcher and providing written informed consent. Patients were informed that they are free to leave the study anytime without any effect on their treatment plan should they wished to do so. All methods were performed in accordance with the relevant guidelines and regulations, which are aligned with the Declaration.

## Data availability statement

The datasets generated in the current study are available from the corresponding author upon reasonable request.

## **Declaration of Generative AI and AI-assisted technologies in the writing process**

No AI tools/services were used during the preparation of this work.
